# Critical deposition height for sustainable restoration via laser additive manufacturing

**DOI:** 10.1038/s41598-018-32842-z

**Published:** 2018-10-03

**Authors:** Santanu Paul, Ramesh Singh, Wenyi Yan, Indradev Samajdar, Anna Paradowska, Khushahal Thool, Mark Reid

**Affiliations:** 1 Department of Mechanical Engineering, Indian Institute of Technology Bombay, Mumbai, 400076 India; 2 Department of Mechanical and Aerospace Engineering, Monash University, Clayton, VIC 3800 Australia; 3 Department of Metallurgical Engineering and Material Science, Indian Institute of Technology Bombay, Mumbai, 400076 India; 4 Australian Nuclear Science and Technology Organization, New Illawarra Rd, Lucas Heights NSW, 2234 Sydney, Australia

## Abstract

Laser material deposition based restoration of high-value components can be a revolutionary technology in remanufacturing. The deposition process induces residual stresses due to thermomechanical behavior and metallurgical transformations. The presence of tensile residual stresses in the deposited layer will compromise the fatigue life of the restored component. We have developed a novel fully coupled metallurgical, thermal and mechanical (metallo-thermomechanical) model to predict residual stresses and identified a critical deposition height, which ensures compressive residual stresses in the deposited layer. Any lower deposition height will result in tensile residual stresses and higher deposition height will result in excessive dilution (substrate melting). We have validated the model using neutron and micro-focus X-ray diffraction measurements. This study highlights that the critical deposition height corresponds to the minimum cooling rate during solidification. It addresses one of the major outstanding problems of additive manufacturing and paves a way for “science-enabled-technology” solutions for sustainable restoration/remanufacturing.

## Introduction

Transformation of the global industrial ecology is essential to ensure the stability of the climate-sensitive tipping elements of the Earth System^1–5^ while maintaining sustainable industrial growth. For such a transformation, reorganizing the manufactured capital infrastructure towards repair and remanufacture of industrial products will reduce human impact on the environment as well as increase economic growth^4,6–8^. In this regard, the importance of maintenance, repair and overhaul (MRO) in the aerospace and automotive sectors can be understood from the fact that these sectors significantly leverage other sectors of a nation’s economy^9^. Even by conservative estimates ~18% increase by 2024^10^ is predicted for the automotive collision repair industry and aircraft MRO market^10,11^. Central to these efforts for sustainable development in the automotive and aerospace sectors is extending the service life of molds/dies, engine parts and important structural components using energy efficient Directed Energy Deposition (DED) processes such as laser additive manufacturing^12^. Laser additive manufacturing provides numerous advantages over the traditional restoration techniques based on thermal spraying or welding, which are imprecise and *ad hoc*^13–25^. The precise and controlled deposition in these processes produces a relatively narrow dilution and heat affected zone^15–17,19,26^. However, it induces residual stresses (locked in post processing stresses). Note that the nature of the residual stresses (compressive or tensile) is the most important factor affecting the integrity and quality of the processed components as it directly affects the service life^16,17,19,20,26,27^. The residual stresses develop due to strains induced by thermomechanical effects and metallurgical transformations^28–30^. The deposition of the hot molten material on a relatively cold substrate and subsequent conduction-driven cooling results in differential thermal expansion and contraction between the deposited and substrate layers^21,30–33^. The thermomechanical strains depend on the temperature distribution, the coefficients of thermal expansion and the elastoplastic behavior of the clad and the substrate materials. In addition, the high rate of cooling can result in metallurgical transformations which contribute to the development of additional strains due to transformation induced plasticity and volumetric dilation^34–39^. It may be noted that the deposited layer is often the most critical region and tensile residual stresses in this region can aid in crack propagation^17,18,27,30,31,40,41^. One of the greatest challenges in laser additive manufacturing today is to determine a critical deposition height to ensure compressive residual stress in the clad (from the surface of the clad to the clad-substrate interface region).

Finite element (FE) models provide viable alternatives to expensive experiments for predicting the transient temperature field, the residual stresses and the microstructural transformations developed during the process^15–17,19,26,27,35,42–45^. Most of the finite element models reported in the literature for predicting the residual stresses use sequential coupling between the metallurgical and thermomechanical phenomena^37,39,46,47^. It may be noted that a fully coupled model can consider the effect of metallurgical transformations on the in-process stress evolution and can better predict the residual stress profile. Moreover, the existing models do not take into consideration of the shape of the deposited layer. In this work, we have investigated the residual stress distribution across a laser deposited layer on a substrate via a 3D fully coupled metallo-thermomechanical finite element model. Gaussian distribution of powder with uniform intensity moving heat source is considered in this analysis^22,48^.

To experimentally validate the model, laser cladding (deposition) experiments were conducted on H13 tool steel with Crucible Particle Metallurgy (CPM) steel powders with high Vanadium content. It is known that dies and molds of H13 tool steel are repaired using CPM9V^18,22–24,49,50^. The residual stresses predicted by the model have been compared with the results obtained from micro-focus X-ray diffraction for micro-scale local residual stress measurements at IIT Bombay, and neutron diffraction experiments for determining volume averaged residual stresses at ANSTO, Sydney, Australia. The following sections describe the formulation of the fully coupled 3D metallo-thermomechanical model and experimental details.

## Methodology

### Metallo-thermomechanical modeling of residual stresses

**Figure 1:**
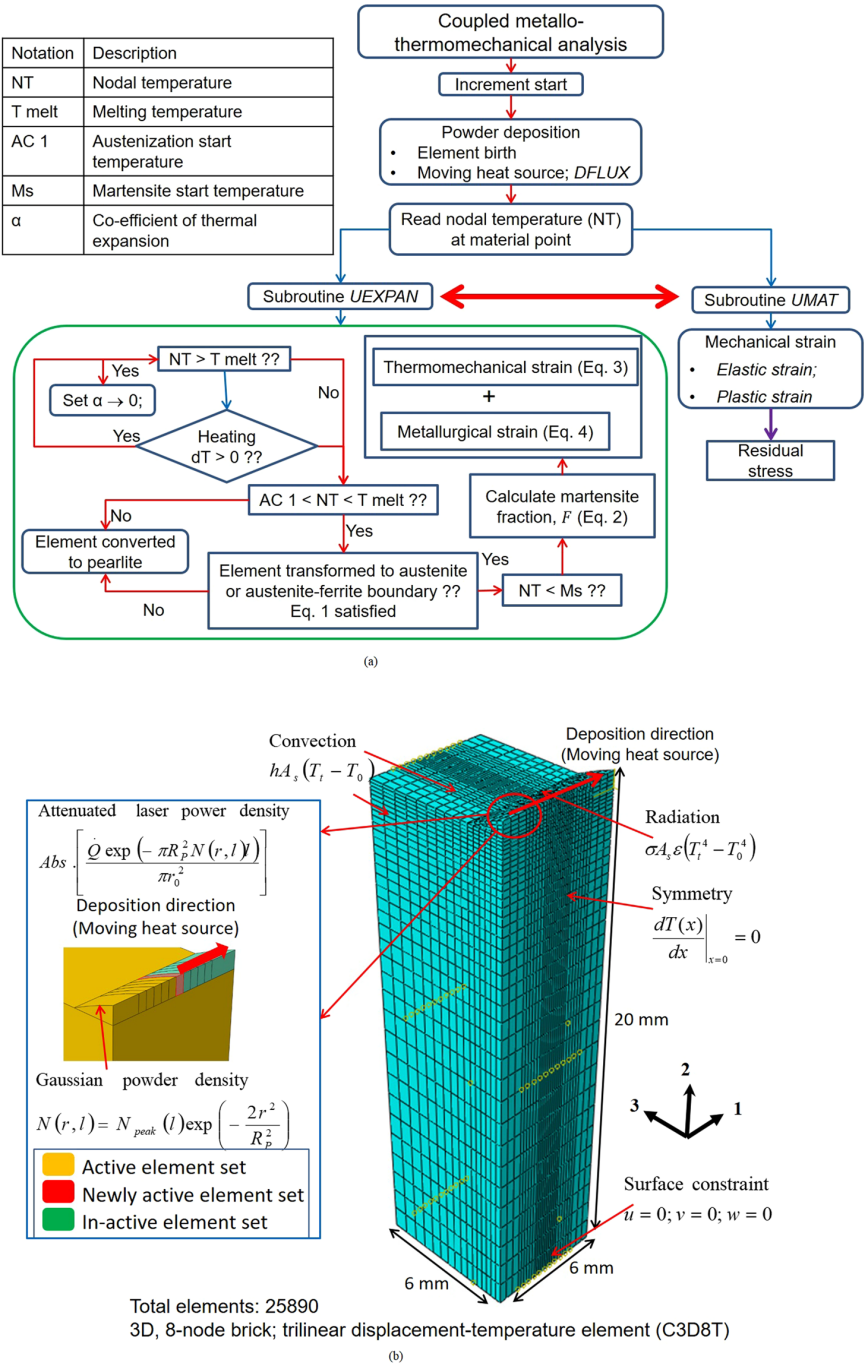
(**a**) Algorithm of metallo-thermomechanical model with (**b**) computational domain.

The 3D fully coupled metallo-thermomechanical finite element model utilizes various user-defined subroutines in the commercial finite element software ABAQUS^®^ to model the complex interactions between the thermal, mechanical and metallurgical phenomena involved in laser cladding. Figure 1 describes the algorithm for the metallo-thermomechanical model along with the computational domain with details of mesh size and loading-boundary conditions. Note that the element activation happens only after the material temperature exceeds the melting temperature. As a result, the clad layer is deposited as a layer of molten material. The coefficient of thermal expansion of the molten material is reduced significantly via a user defined subroutine, so that no further thermal strain is accumulated in the element. Consequently, negligible stresses are present in the clad till the material is molten state (see Fig. 1(a)). To incorporate the strains due to metallurgical transformations, it is crucial to identify the various phase fractions using the kinetic model defined by the multi-utility user subroutine UEXPAN in ABAQUS^®^. The subroutine UEXPAN forms the crucial link between the thermal, metallurgical and mechanical interactions. For the elements that transform to martensite, the volume fraction of martensite (*F*) is calculated according to Eq. (1)^51^ (*for details of governing equations see SI Appendix section* S1).

1 $$F={F}_{m}-({F}_{m}-{F}_{i})exp[\,-\,\frac{12{{F}_{i}}^{2/3}}{\sqrt{\pi }g}ln(\frac{{C}_{e}}{2{C}_{c}})\sqrt{Dt}]$$

where *F*_*m*_ is the maximum fraction of martensite permitted by the phase diagram, *F*_*i*_ is the volume fraction of pearlite, *g* is the grain size, *C*_*e*_ is the carbon content in austenite, *C*_*c*_ is the critical carbon content, D is the appropriate diffusion coefficient for carbon, t is the time required for the lateral diffusion of carbon over a distance λ (pearlite spacing). Apart from calculating *F*, UEXPAN also calculates the strains developed due to differential thermal expansion-contraction $(d{\varepsilon }_{ij}^{th{\rm{\_}}\alpha })$ ^52,53^ and metallurgical transformations $(d{\varepsilon }_{ij}^{TM})$ viz. transformation induced plasticity and volumetric dilation^36,37,42–44^, given by:

2 $$d{\varepsilon }_{ij}^{th{\rm{\_}}\alpha }(T)={\alpha }_{T}dT+(T-{T}_{ref})d{\alpha }_{T}$$

3 $$d{\varepsilon }_{ij}^{TM}=3{K}_{TP}(1-F){S}_{ij}(dF)+\frac{1}{3}{(\frac{{\rm{\Delta }}V}{V})}_{A\to B}d{X}_{p}{\delta }_{ij}$$

4 $$d{\sigma }_{ij}={C}_{ijkl}^{ep}(d{\varepsilon }_{kl}-d{\varepsilon }_{kl}^{p}-d{\varepsilon }_{kl}^{th{\rm{\_}}\alpha }-d{\varepsilon }_{kl}^{TM}+d{C}_{ijkl}^{ep}{\varepsilon }_{kl}^{e})$$

where *K*_*TP*_ = 5.08 × 10^−5^ MPa^−1^ ^37^, ${(\frac{{\rm{\Delta }}V}{V})}_{A\to B}$is the volume change associated from one phase to other, i.e. from ferrite to austenite during heating, and austenite to ferrite or martensite during cooling. *X*_*p*_ corresponds to the resultant phase during the transformation, i.e. austenite during heating and ferrite or martensite during cooling^39^. The actual material behavior of H13 was determined in the thermomechanical simulator Gleeble (Fig. S1) and temperature dependent thermo-physical properties of H13 and CPM 9 V (Table S1, *refer to* Table S2 *for microstructural properties of H13*) have been used in the analysis^54,55^. The user defined subroutine UMAT has been used to define the mechanical constitutive behaviour of the material^56–58^, approximated by a modified Johnson-Cook plasticity model where strain rate hardening was ignored^32,59^ (Fig. S1), to calculate the elastic $({\varepsilon }_{kl}^{e})$, plastic $({\varepsilon }_{kl}^{p})$ strains and elasto-plastic constitutive matrix $(\,{C}_{ijkl}^{ep})$^52,53,60,61^. Mesh convergence study was conducted to understand the behavior of the model for varying mesh sizes. Accordingly, the mesh size reported in Fig. 1(b) is obtained after mesh convergence study (*see* Fig. S3(a)).

### Experimental details

**Figure 2:**
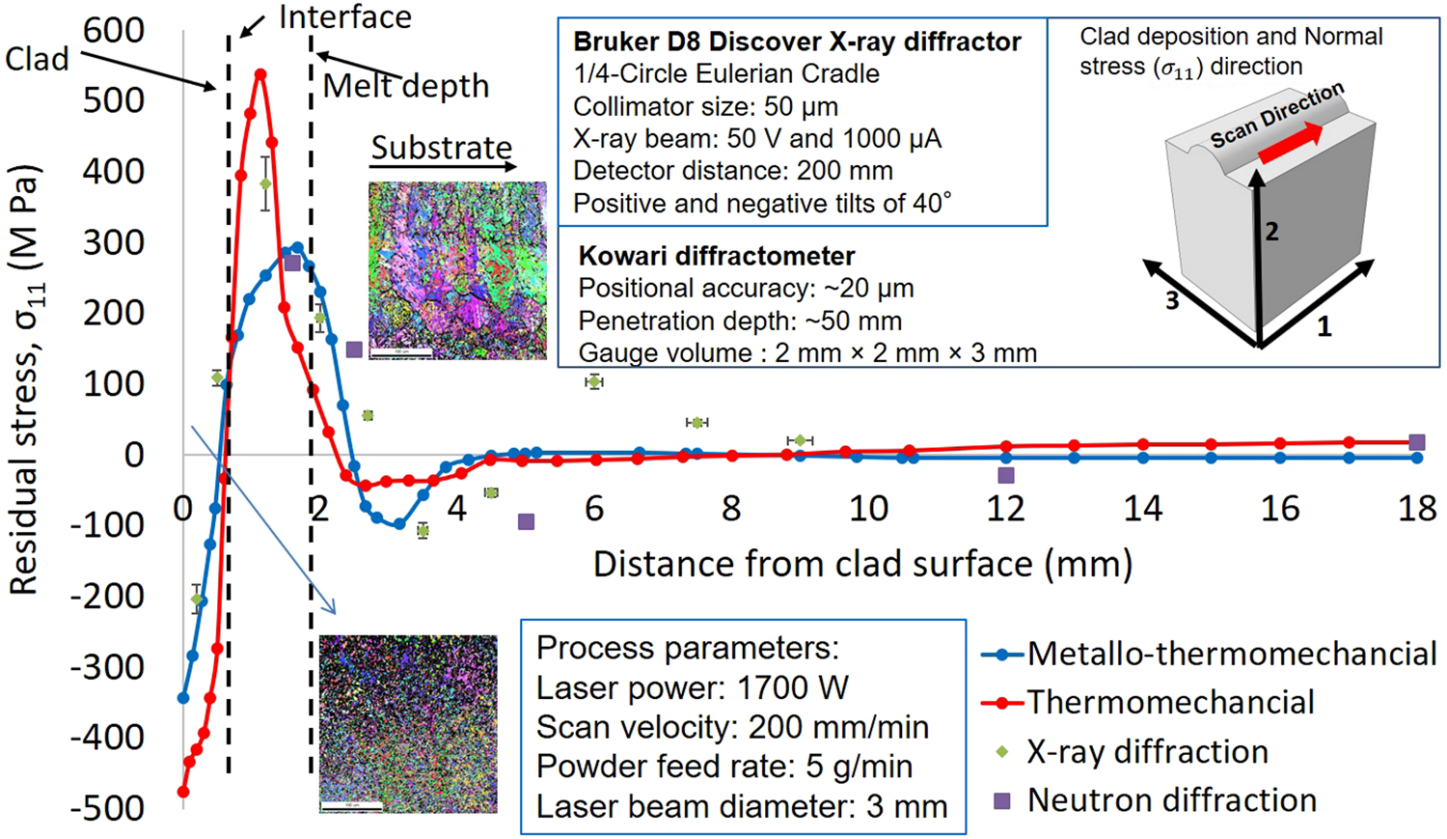
Comparison of residual stress predicted by finite element models with measured using Neutron and X-ray diffraction.

The volume averaged residual stresses in the laser cladded components were measured using Neutron diffraction at the KOWARI residual stress diffractometer at the Australian Nuclear Science and Technology Organization (ANSTO). For measurements, the Fe-211 reflection with neutron wavelength of 1.68 Å was used. The gauge volume for longitudinal measure was fixed at 2 mm × 2 mm × 3 mm. The limitation of the gauge volume used for neutron diffraction measurement restricted the measurement of the macroscale residual stresses to the clad zone. Consequently, the local micro-scale residual stress was measured using the Bruker D8 Discover X-ray diffractometer with a 300 μm spot size. The details of the neutron and micro-focus X-ray diffraction measurements are provided in Fig. 2 (for details of the calculation of residual stresses in diffraction measurements refer to *SI Appendix section* S3).

## Results and Discussion

### Influence of metallurgical transformations

Figure 2 shows a comparison of the normal residual stresses measured using diffraction techniques with the residual stresses predicted by the thermomechanical and the metallo-thermomechanical finite element models. Both the diffraction techniques showed the presence of tensile residual stresses near the melt front and compressive stresses in the deposited layer and the interface regions. The thermal stresses develop primarily due to different coefficients of thermal expansions between the deposited clad and substrate materials^21,32^ and the temperature difference due to the thermal gradient. In laser cladding, the shrinkage of the solidified deposited layer is constrained by the relatively large and cold substrate, which induces tensile stresses at the melt interface^26,32,62^. The force equilibrium and the metallurgical changes result in a complex residual stress profile (see Fig. 2). Figure 2 shows that the thermomechanical model could capture the nature of the residual stresses in the deposited layer and the substrate where the thermomechanical effects due to differential thermal expansion and contraction dominate; but fails to predict the compressive stress in the substrate. This is because the high cooling rates associated with laser cladding results in the formation of metastable martensite phase^32,42,63,64^. On inclusion of the metallurgical effects, the metallo-thermomechanical model can capture the presence of the compressive residual stresses in the substrate. Note that the thermomechanical model considers the stress developed due to thermal strains whereas the metallo-thermomechanical model considers the combined effect of thermal and metallurgical strains for calculation of residual stresses. The prediction error for compressive stresses in the clad layer significantly improves from ~26% for the thermomechanical model to ~2% for the metallo-thermomechanical model. Additionally, the prediction error for the tensile stresses near the melt depth region improves from ~29% to ~13% on considering the metallurgical effects. Additionally, the Electron Backscatter Diffraction (EBSD) images of the microstructures in the clad and substrate region are included in Fig. 2. From Fig. 2, it can be observed that very fine equiaxed alpha phase is present in the clad region with predominantly prior austenitic grain and a lath morphology is present in the substrate region^27,33,63^. Though the effect of the transformation induced plasticity is minor relative to the thermal strains and the volume dilation, the inclusion of metallurgical effects significantly improves the prediction of the residual stresses. Therefore, the subsequent analysis is conducted using the improved metallo-thermomechanical model.

### Critical height of deposition

**Figure 3:**
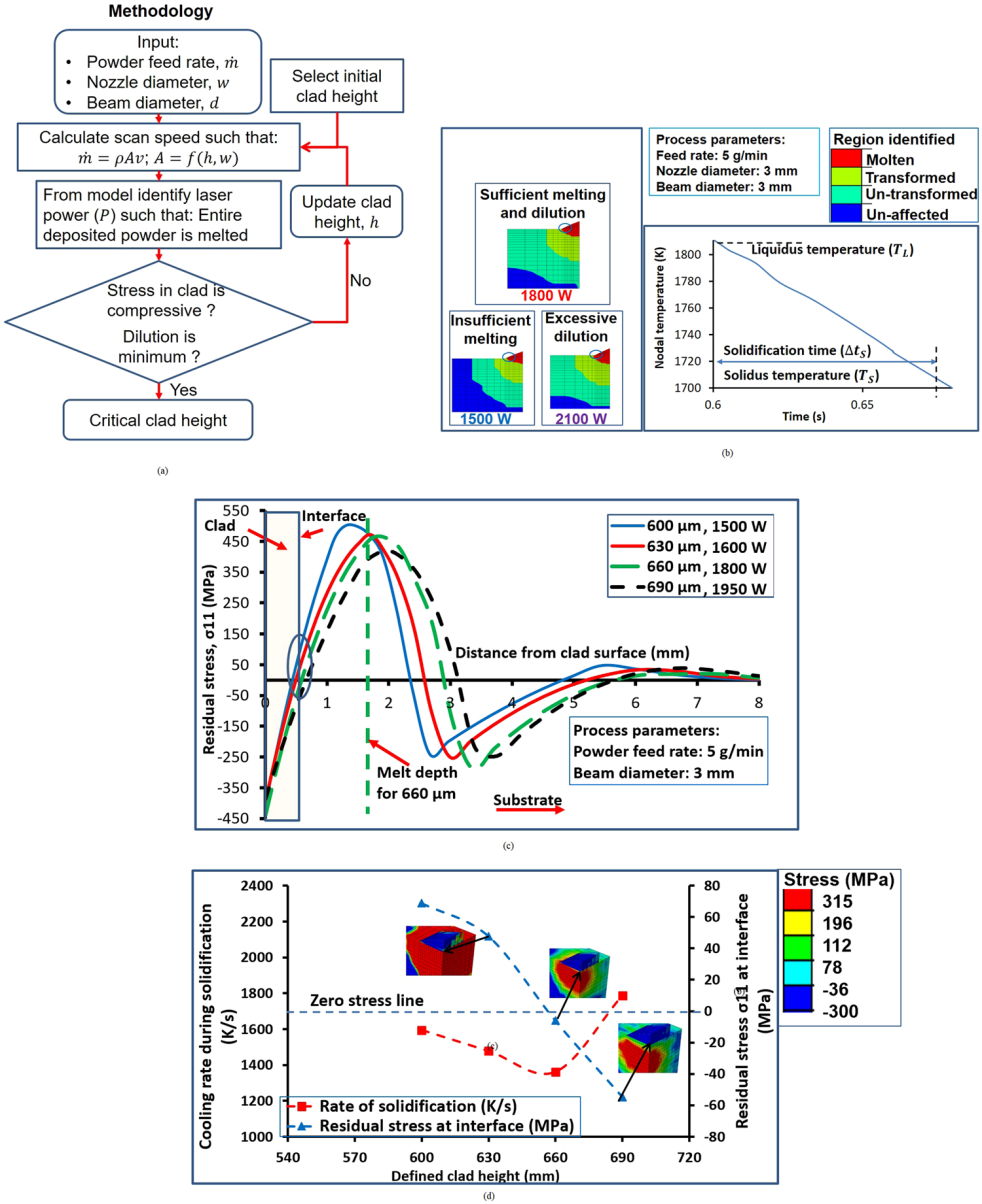
(**a**) Methodology for determining critical height of deposition. (**b**) Dilution and nodal temperature variation. (**c**) Comparison of different clad heights to identify critical height of deposition. (**d**) Rate of solidification and residual stress at interface for different clad heights.

The critical height of deposition corresponds to the layer thickness which when deposited would ensure compressive residual stresses in the deposited layer. An iterative methodology has been devised (see Fig. 3(a)), wherein, for given input process variables, namely, powder feed rate, nozzle and beam diameter, a trial value of clad height and corresponding scanning speed (based on continuity equation for mass conservation as the powder feed rate is fixed) is selected. The next step is to determine the laser power required, to ensure that the entire deposited layer is melted with minimal substrate melting. The predicted residual stress distribution of this combination of trial height and laser power is calculated and checked if the residual stress in the clad layer is compressive and the clad-substrate interface is free of tensile residual stresses. If the above conditions are not satisfied, the trial-clad height is updated and the entire sequence of steps is repeated to identify the critical height of deposition. The converged clad height is termed as critical deposition height where the entire clad is under compressive stresses. Note that in this study, *σ*_11_ (the normal component of residual stress along the direction of deposition) is considered (see Fig. 2). It is important to point out that *σ*_11_ is not the principal stress but the normal residual stress component in the longitudinal (scan) direction. Previous studies have reported that the longitudinal residual stress is the dominant residual stress component for welding and laser assisted deposition operations^41,44,65,66^.

To obtain the critical height from the model proposed in this paper, the trial-clad height is increased from 600 µm to 690 µm for a powder feed rate of 5 g/min with a nozzle diameter and spot size of 3 mm (see Fig. 3(c)). The laser power required for completely melting the deposition height of 600 µm is 1500 W whereas for 690 µm deposition height, the laser power is 2250 W.

It may be noted that the scan speed will change for every trial clad height based on the conservation of mass. Even though a substantial portion of the clad is under compressive residual stresses, the clad-substrate interface is under tensile stresses for 600 and 630 µm deposition heights. However, if the clad height is increased to 660 µm, the residual stresses at the interface also become compressive. If the clad height is increased further to 690 µm, the residual stresses at the interface remain compressive, albeit at a higher magnitude. It may be noted that the melt depth increases substantially resulting in higher dilution in the substrate, which is detrimental. Consequently, 660 µm (at a laser power of 1800 W and a corresponding scan speed of 354 mm/s) has been identified as a critical deposition height, any lower or higher clad heights will result in unfavorable deposition conditions. To understand the physics underlying the residual stress generation, the effect of the cooling rate during solidification has been investigated at different deposition heights. The cooling rate during solidification *R* is given by^63,64,67,68^:

5 $$R=\frac{{T}_{L}-{T}_{S}}{{\rm{\Delta }}{t}_{s}}$$

where *T*_*L*_ is the liquidus temperature (~1811 K), *T*_*S*_ is the solidus temperature (~1700 K)^32^ and Δ*t*_*s*_ is the solidification time obtained from the variation of nodal temperature^32^ (see Fig. 3(b)). It is expected that *R* will govern the residual stress evolution as the onset of thermomechanical residual stresses occurs at solidification. It has been observed that *R* is 1593 K/s at a deposition height of 600 µm. *R* decreases with an increase of deposition height and at 660 µm *R* is 1360 K/s. It may be noted that beyond the critical deposition height, *R* increases to 1786 K/s at 690 µm. Hence, the critical deposition height corresponds to the lowest value of *R* (see Fig. 3(d)). The low cooling rate during solidification (*R*) ensures that the thermomechanical residual stresses are lower.

### Summary and Concluding Remarks

In this work, we investigated the variation of residual stress across the cross-section of a laser cladded specimen, predicted using a fully coupled metallo-thermomechanical model to demonstrate the existence of a critical deposition height. Any deposition height lower than the critical height would yield detrimental tensile residual stresses at the interface whereas higher than the critical deposition height will result in undesired excessive dilution. It was also found that at the critical height of deposition, the solidification rate is minimum. The study also highlights the importance of strains developed due to metallurgical transformation on the final residual stress variation across the cross section of the repaired specimen. This study addresses one of the most important problems in additive manufacturing that whether a critical deposition height exists which can yield compressive residual stresses in the deposited layer and the interface. This study can form a basis of “science enabled technology solutions” for improving the quality of components produced using additive manufacturing techniques. It may be noted that if the restoration is carried out at this deposition height, the service life of the restored component will be enhanced. This could pave the way to sustainable restoration via energy-efficient laser additive manufacturing, thereby optimizing the total material cycles for industrial production.

## Methods

All numerical simulations were performed utilizing various user-defined subroutines (DFLUX, UEXPAN and UMAT) in the commercial finite element software ABAQUS^®^. The subroutines are written to relate to the algorithm described in Fig. 1. Detailed explanation can be found in section ‘Methodology’.

### Code Availability

All numerical codes in this paper are available upon request to the corresponding author.

## Electronic supplementary material

Supplementary Information

## Data Availability

All relevant data in this paper are available from the authors.
