# Supplementary material for: Critical deposition height for sustainable restoration via laser additive manufacturing
Source: Sci Rep. 2018 Oct 3;8:14726. doi: 10.1038/s41598-018-32842-z (PMC6170464; doi:10.1038/s41598-018-32842-z)
Supplement: Supplementary file 1 — Supplementary Information [file 41598_2018_32842_MOESM1_ESM.pdf]

Supplementary Information

Critical deposition height for sustainable restoration via laser additive manufacturing

Santanu Paul[a, b], Ramesh Singh[a*], Wenyi Yan[b], Indradev Samajdar[c], Anna Paradowska[d], Khushahal Thool[c], Mark Reid[d]

[a]Department of Mechanical Engineering, Indian Institute of Technology Bombay, Mumbai, India, 400076
[b]Department of Mechanical and Aerospace Engineering, Monash University, Clayton, VIC 3800, Australia
[c]Department of Metallurgical Engineering and Material Science, Indian Institute of Technology Bombay, Mumbai, India, 400076
[d]Australian Nuclear Science and Technology Organization, New Illawarra Rd, Lucas Heights NSW 2234, Sydney, Australia

*Corresponding author
rsingh@iitb.ac.in

S1 Governing Equations

S1.1 Thermal analysis

In a coupled thermomechanical finite element for the analysis of laser cladding, the values of nodal temperature obtained from thermal analysis are used as input to calculate the mechanical response, particularly the residual stresses developed. Transient heat conduction equation for a homogeneous, isotropic material is used as the basic governing equation for thermal analysis, which is given as[1]:

$$\rho C_p \left(\frac{\partial T}{\partial t} + U\, \nabla T\right) = \nabla(K\, \nabla T) + H \tag{1}$$

The first term on the right side of Eq. (1), refer to the conductive heat transfer. The second term in right side depicts the convective part of heat transfer due to movement of the laser beam with velocity U. The first term on the left side of Eq. (1) depicts the transient nature of the heat transfer process. The top of the material being added at each instant is subjected to a specified heat flux $\dot{Q}$ due to laser beam irradiation (uniform heat source). In this study, a constant absorptivity value of 0.68 has been used[2]. The variation of absorptivity with temperature is beyond the scope of the current work and will be included in future studies. In addition, the width of the deposited clad is assumed to be equal to the powder delivery nozzle diameter. The rest of the work piece surfaces open to atmosphere is subjected to convective and radiative heat losses. These boundary conditions can be stated mathematically as[1]:

$$K\frac{\partial T}{\partial n} + h(T - T_0) + \sigma\varepsilon(T^4 - T_0^4) - Abs.\left[\frac{\dot{Q}\exp(-\pi R_P^2 N(r,l)l)}{\pi r_0^2}\right] = 0 \tag{2}$$

The first term in Eq. (2) represents heat loss due to conduction from the surface whose unit normal is n. The second and third term refers to convection and radiation heat losses from the surface of the work-piece. The fourth term is the attenuated laser power incident on the deposited powder surface[3]. The converged coaxial powder flow $N(r,l)$ can be approximated by a Gaussian distribution[3] given by:

$$2\int_0^L \int_0^{R_P} N_{peak}(l) exp(-\frac{2r^2}{R_P^2})\, h\, dz\, dr = \left(\frac{3r_n^2}{4r_p^3}\right) \times v_{scan} \times \Delta t \tag{3}$$

where N_{peak} is the peak concentration of powder particles at the center of the powder flow (where *r = 0*), and Δt is the powder deposition time calculated for the entire clad length (L). Besides these, the following initial and final conditions are also satisfied:

$$T(x, y, 0) = T_0 = T(x, y, \infty) \tag{4}$$

S1.2 Kinetic analysis

The kinetic model for the transformation of pearlite to austenite used here is based upon the seminal work of Ashby and Easterling[4]. The transformation of pearlite to austenite and the homogenization of carbon in austenite are diffusion controlled. Accordingly, the time for dissolution, where the ends of the carbide plates dissolve, is given by Eq. (5)[4]:

$$L\lambda \geq \int_{t_1}^{t_2} D_0 exp\left(-\frac{Q}{RT(t)}\right) dt \tag{5}$$

where t_1 is the time the AC_1 temperature is reached and t_2 is the current time within the model. Once Eq. (5) is satisfied, the cell is considered to have transformed to austenite or austenite-ferrite boundary, depending on its initial condition. The transformation of austenite to martensite is displacive and occurs at a specific temperature (M_s). After the cell is considered to have transformed to austenite or austenite-ferrite boundary, the volume fraction of martensite is calculated as[4]:

$$F = F_m - (F_m - F_i)exp\left[-\frac{12F_i^{2/3}}{\sqrt{\pi}g} ln\left(\frac{C_e}{2C_c}\right)\sqrt{D\,t}\right] \tag{6}$$

where F_m is the maximum fraction of martensite permitted by phase diagram and is given by:

$$F_m = 0 \;\; if \; T_P < AC_1 \tag{7}$$

$$F_m = F_i + (1 - F_i)\left(\frac{T_P - AC_1}{AC_3 - AC_1}\right) \;\; if \; AC_1 < T_P < AC_3$$

$$F_m = 1 \;\; if \; T_P > AC_3$$

S1.3 Stress analysis

In the metallo-thermomechanical analysis of the process, the effect of metallurgical transformations on total strain is considered. For a small increment, the incremental linear strain tensor is given as[5]:

$$\varepsilon_{ij}^{e} = \varepsilon_{ij}^{e\_old} + d\varepsilon_{ij} - d\varepsilon_{ij}^{p} - \left(d\varepsilon_{ij}^{th\_\alpha} + d\varepsilon_{ij}^{TP} + d\varepsilon_{ij}^{TF}\right) \tag{8}$$

The thermal strain increment due to presence of differential thermal gradient between the clad and substrate is given by[5]:

$$d\varepsilon_{ij}^{th\_\alpha}(T) = \alpha_T \, dT + \left(T - T_{ref}\right) d\alpha_T \tag{9}$$

Transformation induced plasticity is the apparent increase in ductility accompanying austenite-martensite transformation during temperature dependent laser-based deposition techniques such as, laser cladding. The strain developed due to transformation-induced plasticity is modeled as an additional source of strain in the material and the component wise effective transformation plasticity strain ε_{ij}^{TP} is culated as[6]:

$$\varepsilon_{ij}^{TP} = 3K_{TP}F(1-F)S_{ij}(dF) \tag{10}$$

where $K_{TP} = 5.08 \times 10^{-5}$ MPa^{-1} [6]. The volume dilation component induces thermal strain increment of 4.4 % associated with martensite formation and is given by[2,6]:

$$\varepsilon_{ij}^{TF} = \frac{1}{3}\left(\frac{\Delta V}{V}\right)_{A\to B} X_p \delta_{ij} \tag{11}$$

where $\left(\frac{\Delta V}{V}\right)_{A\to B}$ is the volume change associated from one phase to other i.e. from ferrite to austenite during heating, and austenite to ferrite or martensite during cooling. X_p corresponds to the resultant phase during the transformation i.e. austenite during heating and ferrite or martensite during cooling[2,6]. The final stress σ_{ij} and elastic strains are connected through elastic constitutive matrix C_{ijkl}^{e} given by Eq. (12) as [7, 8]:

$$d\sigma_{ij} = C_{ijkl}^{ep}\left(d\varepsilon_{kl} - d\varepsilon_{kl}^{p} - d\varepsilon_{kl}^{th_{\alpha}} - d\varepsilon_{kl}^{TP} - d\varepsilon_{kl}^{TF} + dC_{ijkl}^{ep}\varepsilon_{kl}^{e}\right) \tag{12}$$

Such that $dC_{ijkl}^{e}\varepsilon_{kl}^{e} = \frac{d\mu}{\mu}S_{ij} + \frac{\sigma_{kk}}{3}\frac{dK}{K}\delta_{ij}$. μ and K are the Lame's constant. The plastic strain increment ($d\varepsilon_{ij}^{p}$) can be expressed by the associative flow rule as[7,8]:

$$d\varepsilon_{ij}^{p} = \Lambda\frac{\partial f}{\partial \sigma_{ij}} = \Lambda\left(S_{ij} - \alpha_{ij}\right) = \frac{3}{2\sigma_Y}d\Lambda\left(S_{ij} - \alpha_{ij}\right) \tag{13}$$

where the plastic multiplier, Λ is a positive scalar which depends on the deviatoric stresses (S_{ij}) and plastic strain increments.

$$d\Lambda = \frac{a_{ij}C_{ijkl}^{e}\left[d\varepsilon_{kl} - d\varepsilon_{kl}^{th}\right] - \left(\frac{\partial \sigma_{Y0}}{\partial T} + (1-\gamma)\frac{\partial H}{\partial T}\bar{\varepsilon}^{p}\right)dT - a_{ij}S_{ij}\left(\frac{\partial \mu}{\mu}\right)}{H + a_{ij}C_{ijkl}^{e}a_{kl}} \tag{14}$$

where $a_{ij} = \frac{3}{2\sigma_Y}\left(S_{ij} - \alpha_{ij}\right)$, $H = \frac{d\sigma}{d\varepsilon^{p}}$ is the strain hardening parameter and σ_{Y0} is the yield strength of the material at room temperature. Substituting from Eq. (13) and (14) to Eq. (12), we have,

$$d\sigma_{ij} = \left(C^{e}_{ijkl} - \frac{C^{e}_{ijkl}a_{kl}a_{ij}C^{e}_{ijkl}}{L}\right)\left(d\varepsilon_{kl} - d\varepsilon^{th}_{kl}\right) - \left(\frac{a_{ij}C^{e}_{ijkl}a_{kl}S_{ij}}{L}\right)\left(\frac{\partial\mu}{\mu}\right) - \frac{C^{e}_{ijkl}a_{kl}}{L}\left[\frac{\partial\sigma_{Y0}}{\partial T} + (1-\gamma)\frac{\partial H}{\partial T}\bar{\varepsilon}^{p}\right]dT + dC^{e}_{ijkl}\varepsilon^{e}_{kl} \tag{15}$$

Rearranging, the stress-strain constitutive relation can be rewritten as[7,8]:

$$d\sigma_{ij} = C^{ep}_{ijkl}\left(d\varepsilon_{kl} - d\varepsilon^{th}_{kl}\right) + dM_{ij} \tag{16}$$

where C^{ep}_{ijkl} is the elasto-plastic tangent matrix. Such that,

$$\begin{aligned} C^{ep}_{ijkl} &= C^{e}_{ijkl} - \frac{C^{e}_{ijkl}a_{kl}a_{ij}C^{e}_{ijkl}}{L} \\ dM_{ij} &= \left(S_{ij} - \frac{a_{ij}C^{e}_{ijkl}a_{kl}S_{ij}}{L}\right)\left(\frac{\partial\mu}{\mu}\right) - \frac{C^{e}_{ijkl}a_{kl}}{L}\left[\frac{\partial\sigma_{Y0}}{\partial T} + (1-\gamma)\frac{\partial H}{\partial T}\bar{\varepsilon}^{p}\right]dT + \frac{\sigma_{kk}}{3}\left(\frac{dK}{K}\right)\delta_{ij} \end{aligned} \tag{17}$$

S2 Material properties

S2.1 Thermo-physical properties for thermal and stress analysis

Temperature dependent thermo-physical properties, such as thermal conductivity, density and specific heat, have been used in the simulations to determine the transient temperature field. The thermo-physical properties of CPM 9V and H13[9,10] used in the simulation are listed in Table S1. The temperature dependent thermophysical properties of CPM 9V crucible steel powder were obtained from the validated thermal model in a previous study by the same research group. Elastic properties for CPM 9V and H13 are also listed in Table S1.

Table S1 Thermo-physical properties of CPM 9V and H13[9,10]

CPM 9V				
Temperature (K)	Conductivity (W/m^2-K)		Thermal expansion co-efficient	1.19×10^{-5}
293	20.48		Density (kg/m^3)	7455
373	21.6		Specific heat (J/kg-K)	460
573	25.25		Young's modulus (GPa)	221
813	26.08		Poisson's ratio	0.28
H13				

Temperature (K)	Conductivity (W/m^2-K)	Density (kg/m^3)	Thermal expansion co-efficient	Young's modulus (GPa)
293	25	7800		210
673	29	7700	1.26×10^{-5}	180
1383	30	7600	1.32×10^{-5}	140
Poisson's ratio	0.3		Specific heat (J/kg-K)	430

The actual material behaviour of H13 was measured in the Gleeble 3800 thermal and mechanical testing system at IIT Bombay with maximum capacity of 10 kN. The mechanical behaviour was measured at minimum available strain rate of 0.01/s. Figure S1(a) shows the mechanical behaviour for H13 tool steel at different temperatures. Figure S1(b) shows the variation of hardness of CPM 9V and H13 with temperature. As the there is a good relationship between hardness value (H_v) and yield strength for carbon and alloy steels ($\bar{\sigma}$) given by $H_v \approx 3\bar{\sigma}$. So accordingly, the mechanical behaviour of CPM 9V was also assumed to be same as that of H13 at lower temperatures (<1073 K). However, at higher temperatures the behaviour of H13 was almost perfectly plastic as evident from Figure S1(a). So, perfectly plastic behaviour was assumed for both CPM 9V and H13 at higher temperatures considering the difference in hardness value in Figure S1(b). The mechanical behavior measured using Gleebel 3800 was approximated by Johnson-Cook plasticity model with von Mises yield criterion and the yield stress ($\bar{\sigma}$) is assumed to be of the form:

$$\bar{\sigma} = (A + B\bar{\varepsilon}^{a})\left(1 + C\,ln\left(\frac{\dot{\bar{\varepsilon}}}{\dot{\bar{\varepsilon}}_0}\right)\right)\left[1 - \left(\frac{T - T_0}{T_m - T_0}\right)^{b}\right] \quad (18)$$

where, A is yield strength, B is the hardening modulus, C is the strain rate sensitivity coefficient, $\dot{\varepsilon}$ is the equivalent strain rate, $\dot{\bar{\varepsilon}}_0$is the reference strain rate, a is the hardening coefficient, b is the thermal softening coefficient, T_m is the melting temperature.

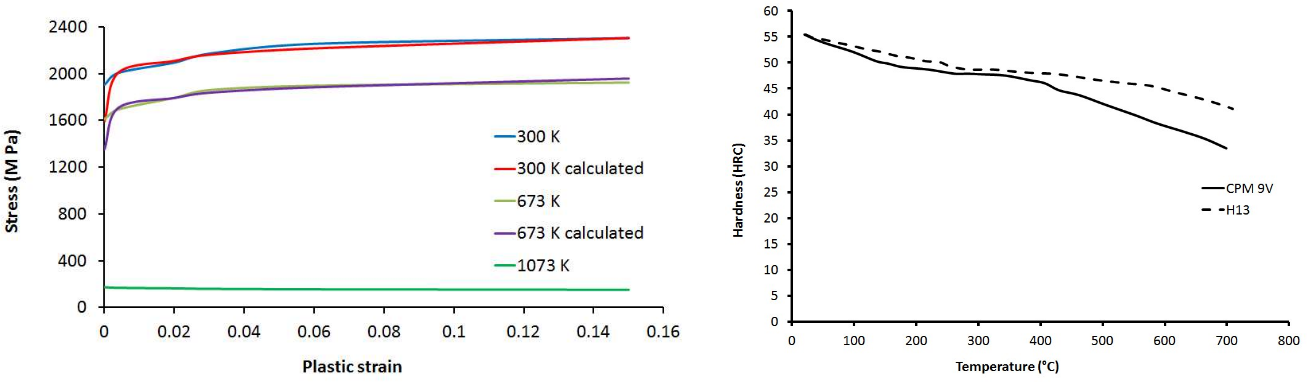

(a) (b)

Figure S1 (a) Plastic strain vs stress variation for H13 tool steel at different temperatures, (b) Variation of hardness of CPM 9V and H13 with temperature, (c) Variation of stress for different mesh sizes

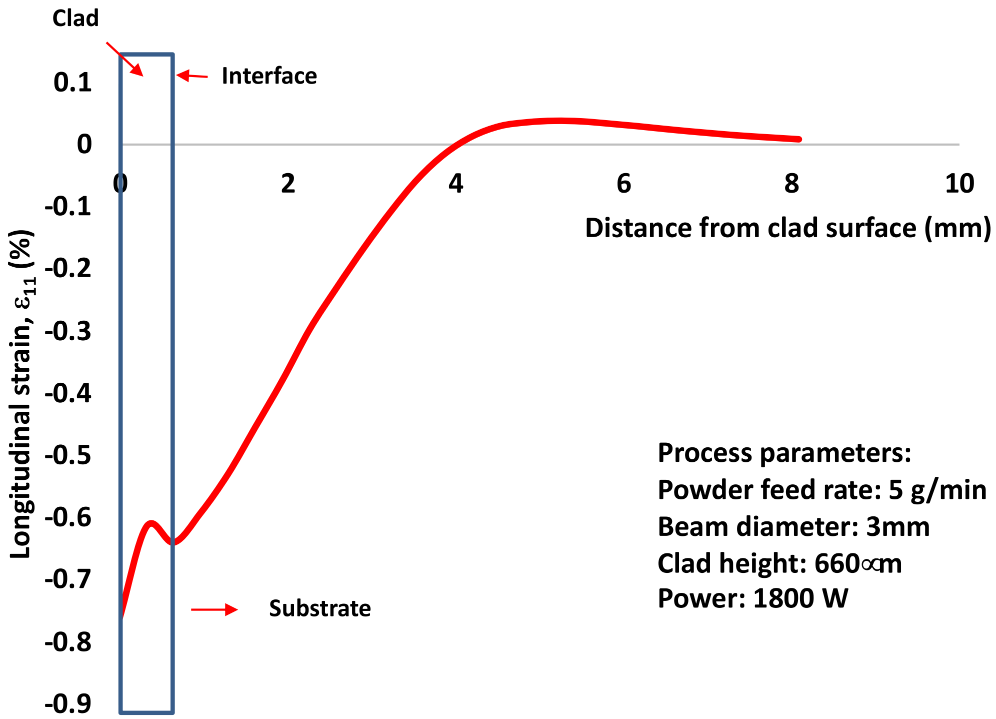

Figure S2 Variation of total strain (in the scan direction) along distance from surface of the clad

Latent heat is generated due to phase transformation, which leads to change of the temperature within the material. The following enthalpy changes proposed by Darken and Gury[11] for the diffusional transformation and Ericsson *et al.*[12] for the diffusion less transformation was used in this work, respectively.

$$\Delta H_P = 953\ kJkg^{-1}K^{-1}$$
$$\Delta H_F = 1769\ kJkg^{-1}K^{-1} \quad (19)$$
$$\Delta H_M = 640\ kJkg^{-1}K^{-1}$$

where ΔH_P, ΔH_F and ΔH_M indicate enthalpy changes according to the temperature for austenite-pearlite, austenite-ferrite and austenite-martensite transformations in that order. Figure S2 shows the variation of percentage longitudinal (in the scan direction) strain, ε_{11} (%) across the cross-section of a laser cladded specimen. The clad and part of the substrate are induced with compressive longitudinal strains. High compressive strains appear at the surface position of the clad which monotonically decrease towards the substrate except a sudden increase near the interface is observed. The maximum value of ε_{11} is ~0.7% (0.007) is not very high and hence the small strain formulation (additive decomposition) has been used in this work.

S2.2 Microstructural properties for kinetic analysis

The metallo-thermomechanical model predicts the athermal martensite transformation in austenite to calculate the volume fraction of martensite. The microstructural properties of the constituent phases for the parent phases used in the model are listed in Table S2.

Table S2 Microstructural properties for H13[2]

Volume change: ferrite to austenite, $\Delta V/V$	-0.38 %
Volume change: austenite to martensite, $\Delta V/V$	1.026 %
Pre-exponential carbon in ferrite, $D_{o\alpha}$	6×10^{-5} m^2/s
Pre-exponential carbon in austenite, $D_{0\gamma}$	1×10^{-5} m^2/s
Pearlite grain spacing, λ	5×10^{-7} m
Diameter of pearlite grain, $L = g/(2f^{1/3})$	8.85×10^{-6} m
Average grain size, g	1.36×10^{-5} m
Volume fraction of pearlite, f	0.45
Critical carbon content value, c_c	0.05 %

S3 Numerical formulation

For the simulations, a half-symmetric model with dimension 6 mm × 6 mm × 20 mm has been developed. Three-dimensional, 8-node thermally coupled brick; trilinear displacement and temperature element (C3D8T) is used. As the variation of the stresses around the clad-substrate interface region is critical. So, in the substrate minimum mesh size of 46µm × 100µm × 100µm is used around the interface with progressively increasing mesh towards the bottom. However, in the clad, uniform mesh of 54µm × 100µm × 100µm has been considered along the cross-section of the clad. The total number of nodes and elements was 28644 and 25890, respectively, which was obtained after mesh sensitivity analysis. Various mesh sizes were analysed to identify the optimal mesh size. In the mesh sensitivity analysis, the variation of secondary variable (stress in this case) for different mesh sizes was compared to ensure the computational efficiency of the current model. The description of the mesh is given in Figure S3(a). Comparing the computational time and the variation of stress variation across the cross section from Figure S3(a) optimal mesh size of Mesh #4 was considered for the analysis.

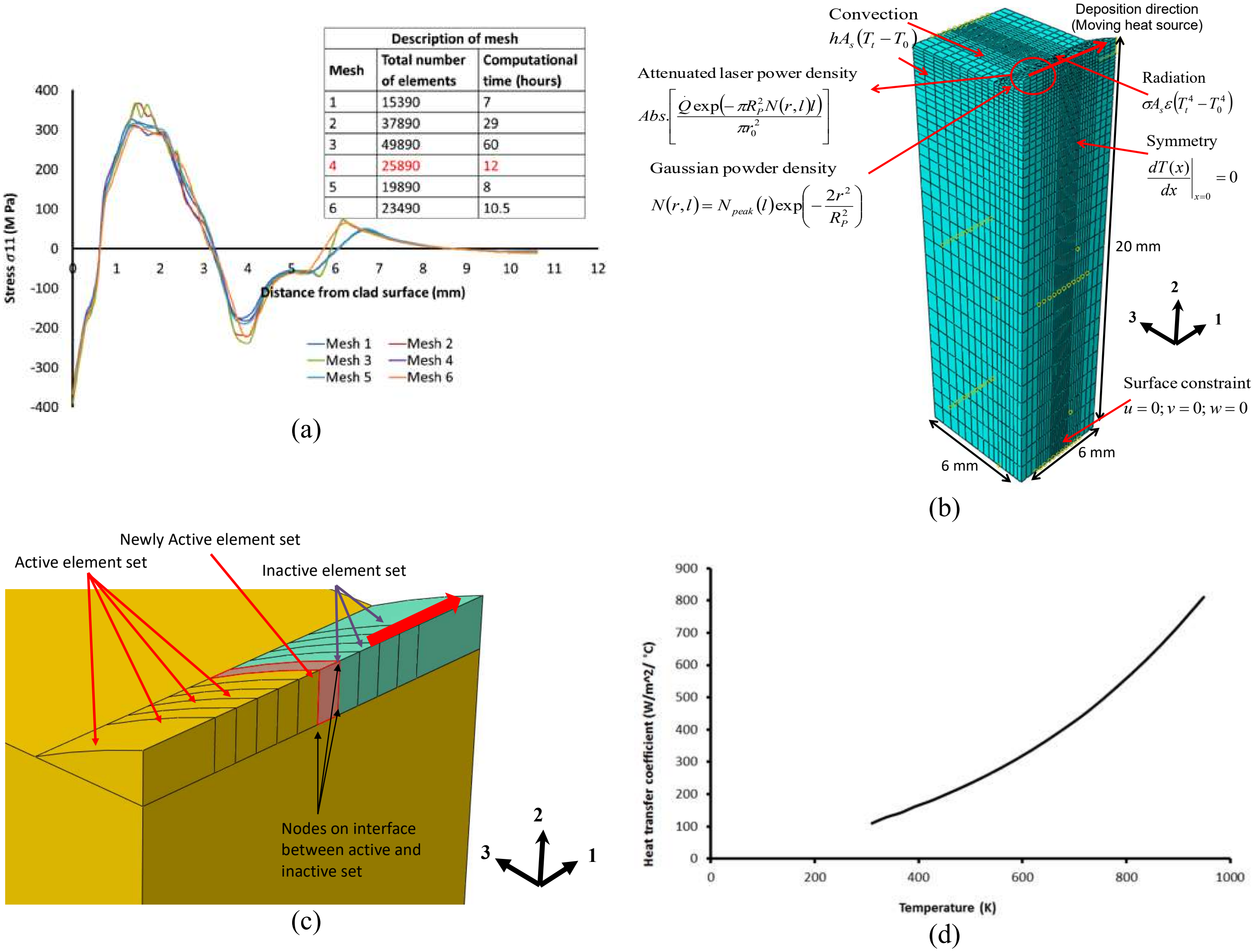

Figure S3 (a) Variation of stress for different mesh sizes, (b) Computational domain showing loading and boundary conditions, (c) Illustration of interface between active and inactive elements conditions, (d) Variation of heat transfer coefficient with temperature[13]

S4 Loading and boundary conditions

Figure S3(b) shows the computational domain along with the loading and boundary conditions. Element birth using the inactive element technique was used to simulate the effect of continuous addition of material on to the substrate during laser cladding operation as shown in Figure S3(c). The temperature of the nodes on the interface between the active and inactive element set are linearly interpolated prior to activation in the next step. The activation of the cladding elements is accompanied by an input heat flux (using subroutine DFLUX) followed by the melting and vaporization of the clad. The direction of motion of the uniform moving heat source is shown in Figure S3(c). The variation of convective heat transfer coefficient with temperature[13] is considered

and is shown in Figure S3(d). Apart from convective heat transfer, radiative heat transfer (with emissivity 0.3) results in heat losses from surfaces. The surfaces subjected to convective and radiative heat losses are shown in Figure S3(b). Apart from the thermal boundary conditions, the mechanical constraints are also shown in Figure S3(c).

S5 Measurement of residual stresses

The residual stress in the laser cladded components were measured using Neutron diffraction at the KOWARI residual stress diffractor at the Australian Nuclear Science and Technology Organization (ANSTO). For measurements, the Fe (211) reflection with neutron wavelength of 1.68Å was used. The gauge volume for longitudinal measure was fixed at 2 mm x 2 mm x 3 mm. In a neutron diffraction analysis, the residual stress and uncertainty in residual stress are calculated from the micro-strains as[14]:

$$\sigma_{11} = \frac{E}{(1+\nu)(1-2\nu)}\left[(1-\nu)\varepsilon_{11} + \nu(\varepsilon_{22}+\varepsilon_{33})\right] \tag{20}$$

$$\Delta\sigma_{11} = \frac{E}{(1+\nu)}\sqrt{\left(\frac{\nu}{1-2\nu}\right)\Delta\varepsilon_{11}^2 + \left(\frac{\nu}{1-2\nu}\right)^2\left(\Delta\varepsilon_{11}^2 + \Delta\varepsilon_{22}^2 + \Delta\varepsilon_{33}^2\right)}$$

The micro-strains and uncertainty in micro-strains are calculated as:

$$\varepsilon_{11} = \frac{d_{11} - d_0}{d_0} \tag{21}$$

$$\Delta\varepsilon_{11} = \frac{d_{11}}{d_0}\sqrt{\left(\frac{\Delta d_{11}}{d_{11}}\right)^2 + \left(\frac{\Delta d_0}{d_0}\right)^2}$$

On the other hand, the calculation of micro-strains is based on the calculation of strain-free lattice spacing for $(h\ k\ l)$ planes, given by:

$$d_0 = \frac{(1-\nu)d_{11} - \nu(d_{11}+d_{33})}{1+\nu} \tag{22}$$

$$\Delta d_0 = \frac{(1-\nu)\Delta d_{11} - \nu(\Delta d_{22}+\Delta d_{33})}{1+\nu}$$

The limitation of the gauge volume used for neutron diffraction measurement restricted measurement of residual stress in the clad zone. Additionally, to measure the variation of local residual stress in the normal direction across the cross section of the laser cladded specimen, micro focus X-ray diffraction analysis was conducted. Accordingly, the residual stresses in the clad, interface and substrate region of the laser cladded components were measured using Bruker D8 Discover X-ray diffractor equipped with a 1/4-Circle Eulerian Cradle with X-ray beam of 50 V

and 1000µA. For residual stress measurements using XRD, the size of the collimator used was 300 µm, as lower spot size did not provide adequate reflection. The detector used was Vantec-500 2D with a detector distance of 200 mm.

The stress distribution in the longitudinal direction (σ_{11}) across the cross-section of the laser cladded specimen was determined by measuring the residual stresses at different spatial locations (represented by dots) in the sample shown in Figure S4. The schematic diagram in Figure S4 clearly shows how the sample was cut-out from the cladded specimen.

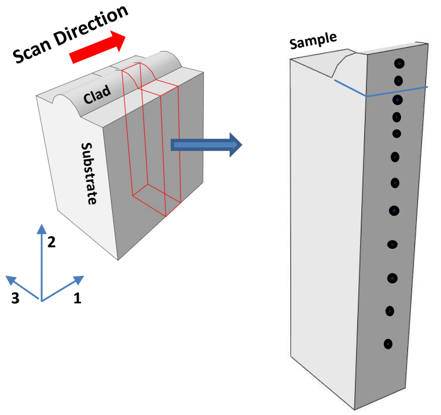

Figure S4 Sample used for measuring residual stresses

It is to be noted that a micro-focus X-ray diffraction unit (with laser-video tracker for identifying exact location of the incident X-ray) was used. For each location (represented by dots in Figure S4), 500 µm oscillation was given in direction 1 (scan direction). An area detector (VantecTM) was used to acquire reliable 2-dimensional X-ray data which in turn was used to determine the stress values at the desired spatial locations. The in-plane normal stress components were measured and the normal stress component in the scan direction (σ_{11}) at the measured locations have been reported in the results as it is the most critical residual stress component.

Figure S5 (a) show a classical d-sin2ψ plot for the (110) pole. The signal was integrated to obtain intensity versus 2θ plots (Figure S5 (b)). The peak shifts were then automatically identified and the d-sin2ψ were plotted to provide the normal residual stress (σ_{11}).

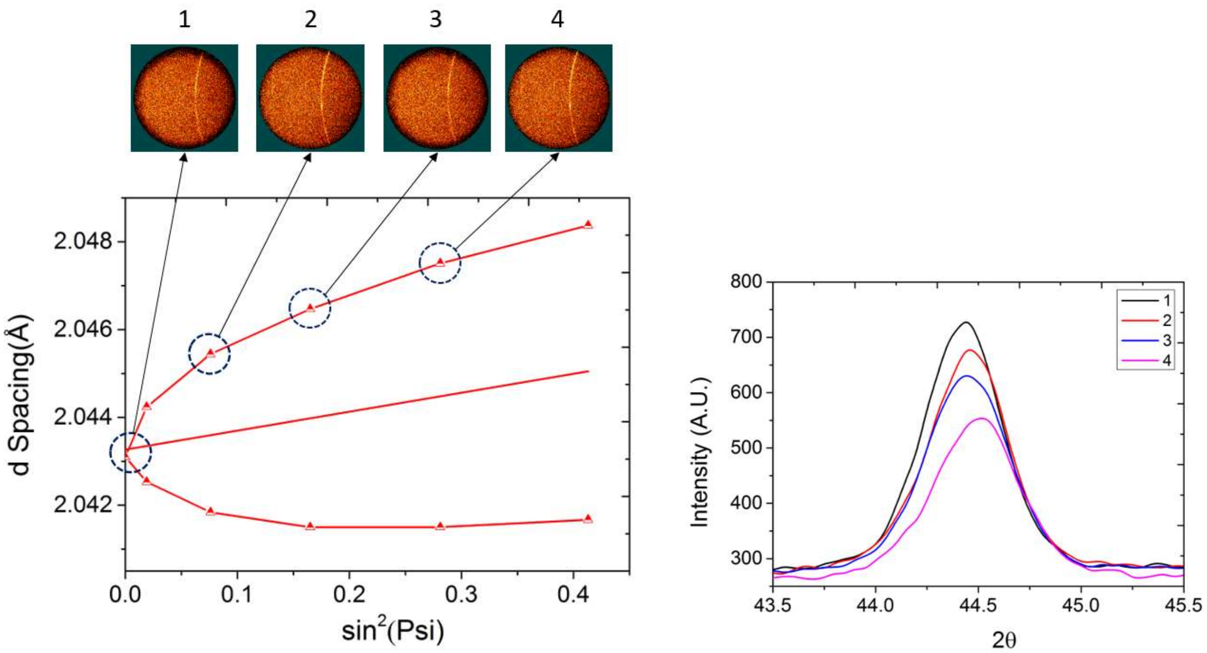

(a) (b)

Figure S5 (a) d-sin$^2\psi$ plot (b) Intensity versus 2θ plots

References

1. Incropera, F. P. & DeWitt, D. P. *Fundamentals of Heat and Mass Transfer.* Water **6th,** 997 (2007).

2. Bailey, N. S., Tan, W. & Shin, Y. C. Predictive modeling and experimental results for residual stresses in laser hardening of AISI 4140 steel by a high power diode laser. *Surf. Coatings Technol.* **203,** 2003–2012, https://doi.org/10.1016/j.surfcoat.2009.01.039 (2009).

3. Qi, H., Mazumder, J. & Ki, H. Numerical simulation of heat transfer and fluid flow in coaxial laser cladding process for direct metal deposition. *J. Appl. Phys.* **100,** https://doi.org/10.1063/1.2209807 (2006).

4. Ashby, M. F. & Easterling, K. E. The transformation hardening of steel surfaces by laser beams—I. Hypo-eutectoid steels. *Acta Metall.* **32,** 1935–1948 https://doi.org/10.1016/0001-6160(84)90175-5 (1984).

5. Paul, S., Thool, K., Singh, R., Samajdar, I. & Yan, W. Experimental Characterization of Clad Microstructure and its Correlation with Residual Stresses. *Procedia Manuf.* **10,** 804–818 https://doi.org/10.1016/j.promfg.2017.07.081 (2017).

6. Ramesh, A. & Melkote, S. N. Modeling of white layer formation under thermally dominant conditions in orthogonal machining of hardened AISI 52100 steel. *Int. J. Mach. Tools Manuf.* **48,** 402–414, https://doi.org/10.1016/j.ijmachtools.2007.09.007 (2008).

7. Neto, E. A. de S., Peric, D. & Owen, D. R. J. *Computational Methods for Plasticity: Theory and Applications*. *Engineering,* John Wiley & Sons, https://doi.org/10.1002/9780470694626 (2009).

8. Hashiguchi, K. & Yamakawa, Y. *Introduction to Finite Strain Theory for Continuum*. John Wiley & Sons. https://doi.org/10.1002/9781118437711 (2013).

9. Xue, L., Chen, J. & Wang, S.-H. Freeform Laser Consolidated H13 and CPM 9V Tool Steels. *Metallogr. Microstruct. Anal.* **2,** 67–78, https://doi.org/ 10.1007/s13632-013-0061-0 (2013).

10. H13 datasheet, Crucible Industries LLC, 575 State Fair Blvd., Solvay, NY 13209, www.crucible.com, 800-365-1180 315-487-4111

11. Stamper Darken, L. & Wilton Gurry, R. *Physical Chemistry of Metals / L.S. Darken, R.W. Gurry.* (2018).

12. Ericsson, T. in *Residual Stresses* 87–113, https://doi.org/10.1016/B978-0-08-034062-3.50015-X (1987).

13. Paul, S., Singh, R. & Yan, W. Thermal model for additive restoration of mold steels using crucible steel. *J. Manuf. Process.* **24,** 346-354 https://doi.org/10.1016/j.jmapro.2016.06.012 (2016).

14. Paradowska, A. *Investigation of Residual Stress in Welds: Using Neutron and Synchrotron Diffraction,. LAP Lambert Academic Publishing AG & CO.KG*, ISBN 978-3-8383-6944-0 *(*2010).
